# A single compartment model to describe lung functionality: A comprehensive study

**DOI:** 10.14814/phy2.70832

**Published:** 2026-03-17

**Authors:** Husam Y. Al‐Hetari, Mahmoud A. Al‐Rumaima, Hamdi H. Ghazi, Noman Q. Al‐Naggar, E. A. Ali, Abdu Alameri

**Affiliations:** ^1^ Department of Biomedical Engineering, Faculty of Engineering University of Science and Technology Sana'a Yemen; ^2^ Department of Biomedical Engineering, Faculty of Engineering and Computing University of Science and Technology Aden Yemen

**Keywords:** lung elastance, lung function, mechanical ventilation, model‐based methods, pressure Control ventilation, respiratory system compliance, single compartment lung model, volume control ventilation

## Abstract

Mechanical Ventilation (MV) is a critical medical intervention used to support patients with impaired lung function caused by severe conditions such as pneumonia or COVID‐19. Model‐based Methods, particularly computational models, are employed to simulate and analyze lung mechanics under MV. Among these, the Single Compartment Lung Model (SCLM) remains the most commonly adopted framework for replicating lung behavior during MV, facilitating optimal treatment strategies. This review critically analyzes existing literatures on SCLM applications, focusing on key parameters such as lung elastance (E), airway resistance (R_rs_), and Dynamic Functional Residual Capacity (dFRC). Methodologies, evaluation metrics, and clinical applications were examined to identify common trends, inconsistences, and research gaps. The findings indicate that E has been the primary focus due to its relevance in assessing lung mechanism, especially under MV. This parameter often evaluated alongside variables like Positive End‐Expiratory Pressure (PEEP), Peak Inspiratory Pressure (PIP), Peak Inspiratory Volume (PIV), and Tidal Volume (V_t_). Additionally, FRC and R_rs_ are also considered in some models. The review emphasizes the need for standardized evaluation protocols, simplified input models, and disease‐specific adaptations to enhance clinical applicability. Our findings provide valuable guidance for future research aiming to refine SCLM‐based approaches and improve personalized mechanical ventilation strategies.

## 1 INTRODUCTION

The respiratory system consists of several organs that function to oxygenate the body through the process of breathing (Aung et al., 2019). The movement of inspired gas into and exhaled gas out of the lung is called ventilation. Understanding lung volumes, respiratory system compliance, and ventilation‐perfusion is essential for the clinical application of respiratory physiology in anesthesia and critical care (Patwa & Shah, 2015).

Mechanical Ventilation (MV) plays a crucial role in the intensive care unit (ICU) to support the respiratory system malfunction by assisting recovery breathing process which could result from diseases and viruses (Al‐Hetari et al., 2024; Al‐Hetari, Kabir, et al., 2020; Gattinoni et al., 2006). The need for MV is one of the most common causes of admission to the intensive care unit (Kalwaje E & Rello, 2018; Metersky & Kalil, 2018). The most common lung diseases, for example, acute respiratory distress syndrome (ARDS), chronic obstructive pulmonary disease, severe asthma, and coronavirus disease (COVID‐19) require MV support in case of higher severity levels. However, incorrect settings of MVs may cause lung injury, therefore, to check the lung functionality and its response to MV, the lung mechanics should be observed while connected to the MV (Al‐Hetari, Alginahi, et al., 2020; Dellamonica et al., 2011).

Recent advances in computational lung modeling have extended beyond reduced‐order representations toward higher‐fidelity approaches that account for regional biomechanics and multiscale mechanical behavior. In particular, artificial intelligence‐assisted multiscale models have been proposed to predict localized alveolar stress and capture spatial heterogeneity in lung mechanics (Neelakantan et al., 2025). Additionally, recent reviews emphasize the growing role of regional biomechanical modeling in enhancing physiological realism in respiratory medicine (Neelakantan et al., 2022).

## 2 BACKGROUND OF SINGLE COMPARTMENT LUNG MODEL

In general, Model‐based methods provide a structured framework for formulating and testing hypotheses while reducing the time, cost, and risk associated with full‐scale experimentation (Kutz, 2009; Thacker et al., 2004). In biomedical and respiratory engineering, models are widely used to analyze complex systems and to simulate airflow, pressure, and volume dynamics in mechanically ventilated lungs (Alginahi et al., 2013; Al‐Hetari, Kabir, et al., 2020; Al‐Naggar, 2015; Kabir et al., 2016; Ouache & Kabir, 2016). Such models enable the estimation of lung mechanics, support clinical decision‐making, and offer noninvasive, continuously updated insight into patient‐specific lung function during mechanical ventilation (MV) (Chase et al., 2006; Chiew et al., 2011; Mishra et al., 2012; Sundaresan et al., 2009; Sundaresan & Chase, 2012; Van Drunen, 2013).

The fundamental model used to represent lung behavior during MV is the Single Compartment Lung Model (SCLM) (Kim et al., 2018). As emphasized in (Neelakantan et al., 2022), computational lung modeling plays a critical role in bridging clinical data with respiratory mechanics. While lung physiology is inherently complex, simplified lumped‐parameter models—specifically the SCLM—remain essential for quantifying global lung behavior. These models provide vital biophysical parameters, including compliance and resistance, which are necessary for developing protective ventilation strategies and preventing lung injury (Neelakantan et al., 2022).

**FIGURE 1:**
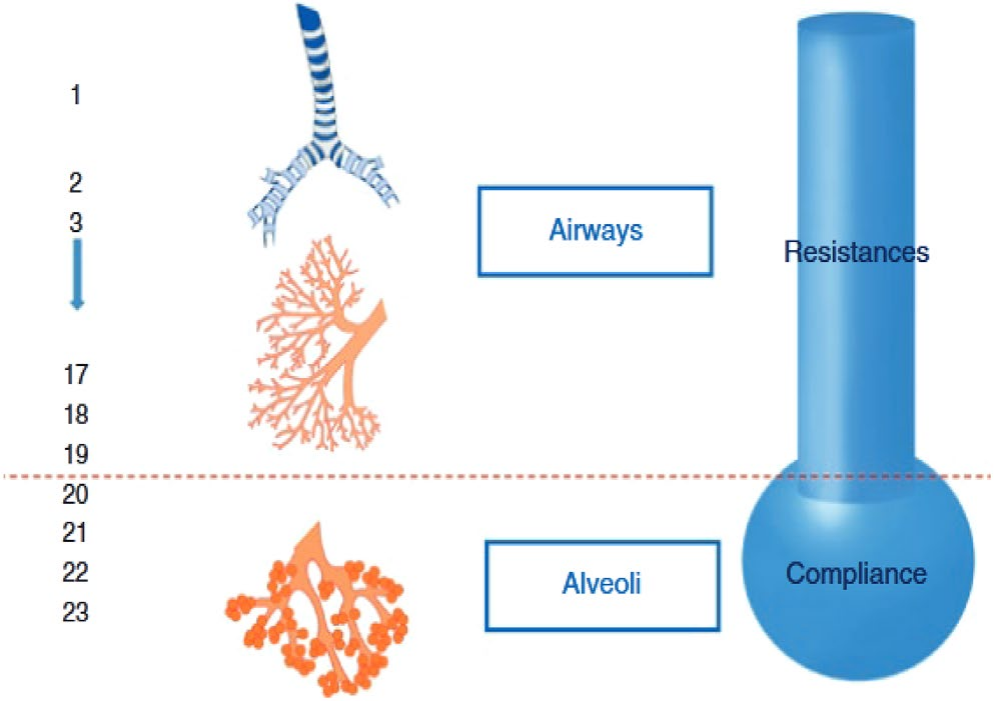
SCLM representation using a pipe (airway resistance) and balloon (lung compliance) (Arnal, 2018).

In SCLM framework, the respiratory system can be simplified to comprise a tube representing the airways that describe airways resistance and a balloon representing the alveoli that describe respiratory system compliance as demonstrated in Figure 1.

A single‐compartment lung model is suitable to mimic lung patients' functionality under MV where it is possible to ignore respiratory system details for compliance and resistance calculations. In clinical practices, most ventilators ignore these details for calculations of resistance and compliance (Chatburn, 2003).

**FIGURE 2:**
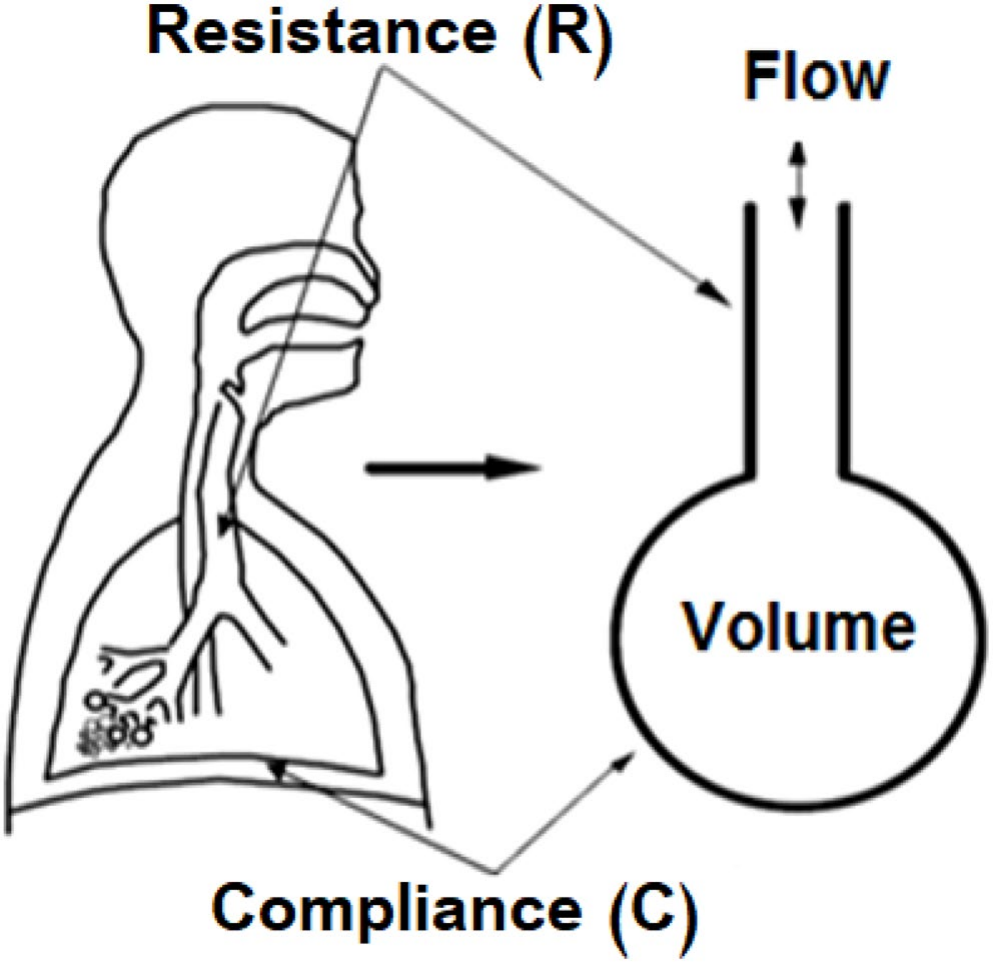
Fundamental representation of a SCLM (Chatburn, 2003).

The SCLM illustrated in Figure 2 is usually used in clinical practice (Chatburn, 2003). This model can simulate the respiratory system as a combination of elastic and resistive components; therefore, the pipe simulates the airway's path of the trachea, and the plastic balloon, which can expand in the same way as the real lung, simulates the lung's compliance.

**FIGURE 3:**
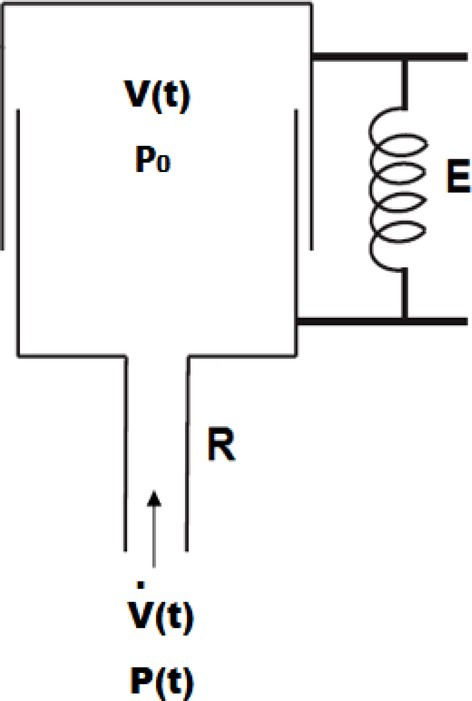
Mechanical schematic of the SCLM (Bates, 2009).

In addition, Bates (2009) used an equivalent mechanical form of this model to describe lung functionality as shown in Figure 3, where the pipe simulates the airway path and the piston connected to a stretched spring simulates the lung which can expand in the same way as the real lung during the inspiration and expiration process.

The P(t) represents the airway pressure, R represents the overall airway resistance, E represents the Elastance, V(t) represents the lung volume, V̇(t) represents the airway flow, P_0_ represents the Positive End‐Expiration Pressure (PEEP) or offset pressure, and t denotes that P, V, V̇ are a function of time.

**FIGURE 4:**
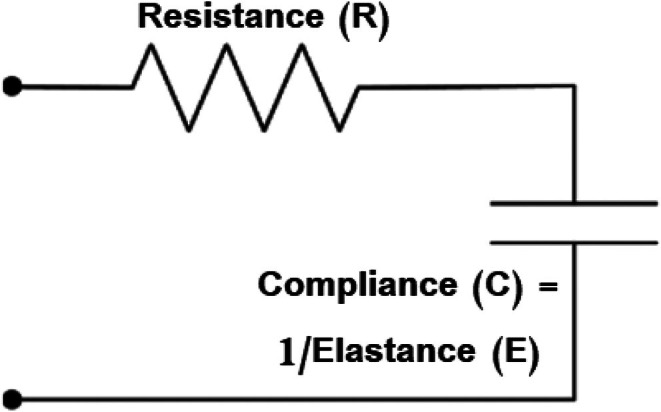
A SCLM as RC circuit (Knörzer et al., 2014).

Furthermore, SCLM can be represented in electrical analogy as a Resistance and Capacitance (RC) circuit (Knörzer et al., 2014) which contains a resistor and a capacitor as shown in Figure 4. In this equivalent electrical model, electrical resistance (R) represents the resistance of airflow, and electrical capacitance (C) represents respiratory system compliance. The current flow (I) simulates airflow, and the voltage source (V_in_) simulates the pressure applied by the ventilator. Physiologically, the mechanical and electrical SCLM representations provide distinct clinical perspectives (Bates, 2009; Knörzer et al., 2014). The mechanical model focuses on structural lung integrity, where the balloon analogy illustrates the physical stiffness of the tissue and the risk of over‐distension. This perspective is vital for determining safe volume limits to protect the lung parenchyma. Conversely, the electrical analogy emphasizes flow dynamics and timing, treating pressure as the driving force and airflow as a moving charge. This representation is indispensable in the ICU for managing the duration of the respiratory cycle to prevent air trapping, a clinical challenge that static mechanical models fail to capture. As highlighted by (Bates, 2009; Knörzer et al., 2014), recognizing these differences allows clinicians to account for the lung's viscoelastic nature and stress relaxation. This comprehensive understanding ensures that ventilator settings are optimized to minimize the risk of ventilator‐induced lung injury.

The basic function of the lung, which is simulated by the SCLM (Arunachalam et al., 2020; Bates, 2009; Chiew, Pretty, Shaw, et al., 2015; Knörzer et al., 2014), is described by Equation (1):

(1) $$P_{\mathrm{aw}}\;\left(t\right)=R_{\mathrm{rs}}\;\dot{V}\left(t\right)+E_{\mathrm{rs}}\;V\left(t\right)+P_{0}$$

This equation considers the main equation of SCLM, where P_aw_(t) is the airway pressure (cmH_2_O), E_rs_ is an overall respiratory system elastance (cmH_2_O/L) (inverse of compliance), V(t) represents lung volume (L), R_rs_ represents airway resistance (cmH_2_O·s/L), $\dot{V}$(t) is air flow (L/s) which equals dV(t)/dt and P_0_ represents PEEP (cmH_2_O). Note that the subscript (t) denotes the time. Many studies used the SCLM equation to improve clinical healthcare for patients who undergo MV therapy.

SCLM parameters can be directly estimated from the model equation: airway resistance (R_rs_) is derived from the pressure–flow relationship, elastance (E_rs_) from the pressure–volume relationship, and PEEP (P_0_) as the baseline offset. These parameters quantitatively capture lung mechanics and can be obtained in real time from measured pressure, flow, and volume signals.

The purpose of this study is to do a comprehensive investigation of the previous works and understand their methodology, assessment criteria, data set, pros, and cons so this study can help to give a complete picture of SCLM which is used to describe lung functionality during MV in critical care, and in future works this can also help to suggest a new idea to reduce the model complexity, cost, etc.

## 3 REVIEW METHODOLOGY

This study presents a comprehensive analysis of existing works that employed Single‐Compartment Lung Model (SCLM) to identify the key parameters characterizing lung functionality during MV and to guide future research.

A total of 31 studies included were identified through systematic searches in major databases (IEEE Xplore, PubMed, ScienceDirect) using keywords such as “Single‐Compartment Lung Model” and “mechanical ventilation,” covering publications from 2003 to 2024. The review methodology was structured into two main components. First, a descriptive summary table was developed for each reviewed study, including the study title, key research elements, strengths, and limitations, providing a clear overview of each study's content and contributions.

Second, four analytical tables were categorized the reviewed studies according to the evaluation metrics used: null hypotheses, fitting error, coefficient of determination (*R* ^2^) and studies using SCLM without formal evaluation metrics. Studies reporting multiple metrics were included in the corresponding tables as appropriate. Each metric category has a distinct purpose: *null hypothesis testing* evaluates changes in physiological parameters (e.g., PEEP effects), *fitting error* quantifies the difference between model predictions and measured data, and *R* ^*2*^ assesses how well the model explains data variability. Each study was examined in terms of data type, ventilation mode, study results, and evaluation methods.

This classification enabled a clear comparison of approaches and findings across previous studies.

## 4 IMPORTANT ASPECTS TO DESCRIBE LUNG FUNCTIONALITY

### 4.1 Elastance (E)

Respiratory system elastance (E), defined as the inverse of compliance, describes the stiffness of the respiratory system and its resistance to deformation during mechanical ventilation. Elastance is an important clinical parameter used to assess the patient's response to MV in the intensive care unit (ICU) (Arunachalam et al., 2020; Chiew et al., 2012; Khirani et al., 2010).

For example, patients with ARDS typically exhibit high elastance (i.e., low compliance) compared to healthy individuals, reflecting increased respiratory system stiffness and impaired lung mechanics (Force et al., 2012).

Real‐time monitoring of respiratory system elastance provides valuable insight into lung mechanics and patient–ventilator interaction. An optimal response to MV is generally achieved when ventilation occurs at lower elastance (higher compliance), allowing greater tidal volume changes and improved oxygenation at lower airway pressures (Lambermont et al., 2008; Suarez‐Sipmann et al., 2007).

#### 4.1.1 Dynamic elastance

**FIGURE 5:**
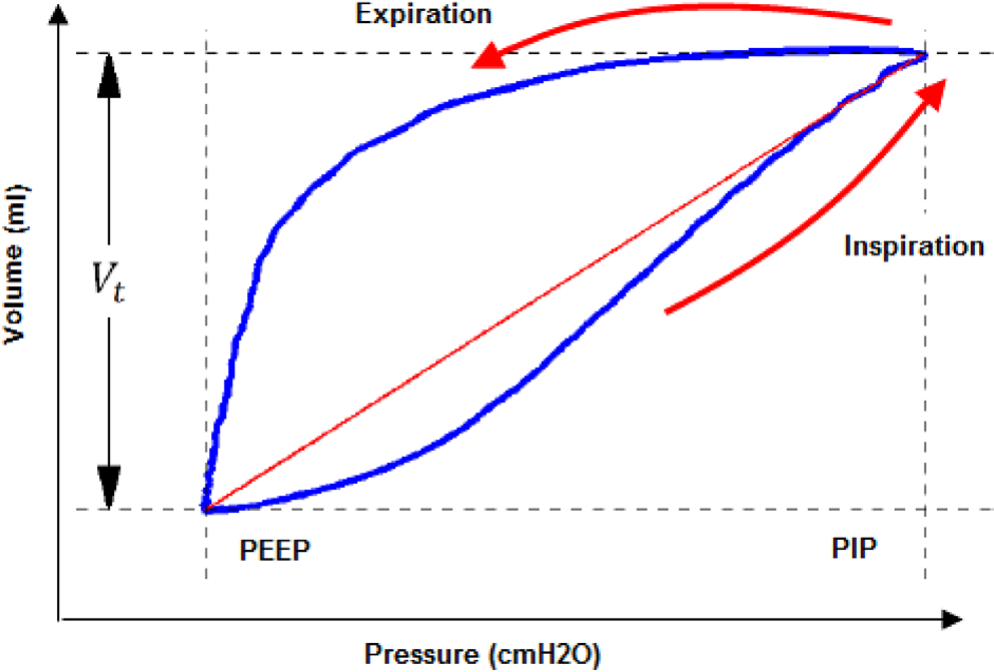
Lung dynamic elastance loop (Van Drunen, 2013).

Dynamic elastance (E_dynamic_) can be estimated from the dynamic Pressure‐Volume (PV) loop as shown in Figure 5. The inspiration phase is represented by the red line, while the expiration phase appears above it. E_dynamic_ depends on PEEP and PIP values, which can be measured at the upper and lower ends of the red line. As shown in Equation (2), dynamic elastance is calculated using the inspired tidal volume (V_t_), PEEP, and PIP (Barberis et al., 2003; Lucangelo et al., 2007).

(2) $$E_{\text{dynamic}}=\frac{\mathrm{PIP}-\text{PEEP}}{V_{t}}$$

The corresponding dynamic compliance (C_dynamic_) can then be calculated as the inverse of dynamic elastance:

(3) $$C_{\text{dynamic}}=\frac{1}{E_{\text{dynamic}}}$$

#### 4.1.2 Static Elastance

Understanding the concept of plateau pressure (P_plat_) is essential. P_plat_ is measured during the end‐inspiration pause (EIP) before the expiration phase when airflow is zero (Fuleihan et al., 1976). P_plat_ is a key measurement for estimating static respiratory system elastance (E_static_). However, when the EIP duration is too short, accurate estimation of E_static_ may be difficult because P_plat_ cannot be reliably captured (Barberis et al., 2003).

Equation (4) shows that the E_static_ can be calculated using V_t_, PEEP, and P_plat_.

(4) $$E_{\text{static}}=\frac{P_{\text{plat}}-\text{PEEP}}{V_{t}}$$

The corresponding static compliance (C_static_) can be calculated as the inverse of E_static_:

(5) $$C_{\text{static}}=\frac{1}{E_{\text{static}}}$$

**FIGURE 6:**
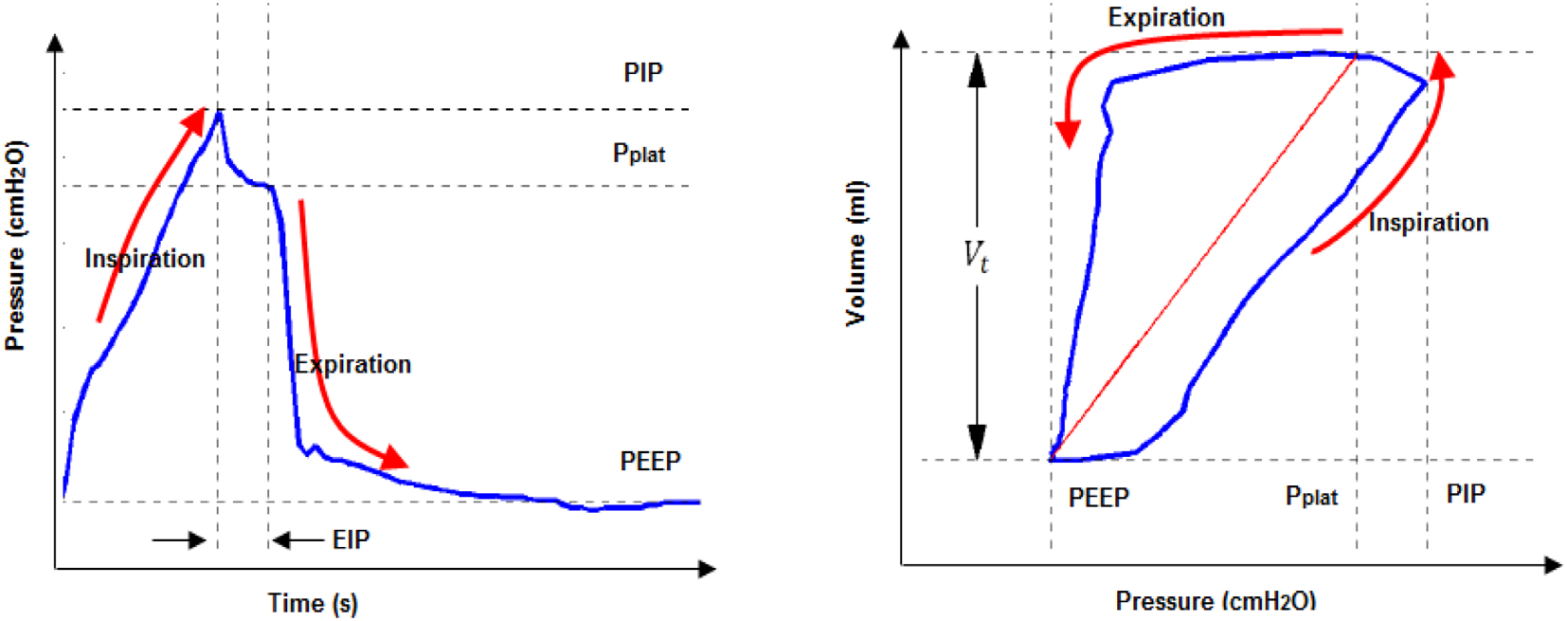
(Left) Pressure signal with P_plat_. (Right) lung static elastance loop (Van Drunen, 2013).

Accordingly, static elastance (E_static_) depends on PEEP and plateau pressure (P_plat_) (Fuleihan et al., 1976). A complete pressure signal of inspiration and expiration phases, including P_plat_, PEEP, and EIP, is shown in Figure 6 (left), whereas Figure 6 (right) illustrates the E_static_ loop, where the inspiration phase appears under the red line and the expiration phase appears above it (Van Drunen, 2013).

### 4.2 Peep and respiratory system elastance

Setting PEEP to achieve minimum respiratory system elastance during MV is considered a useful clinical strategy (Lichtwarck‐Aschoff et al., 2000; Stenqvist & Odenstedt, 2007). Monitoring respiratory system elastance as a function of PEEP can provide valuable clinical guidance for optimizing MV settings and improving patient–ventilator interaction (Chiew, Pretty, Shaw, et al., 2015).

### 4.3 Peep and functional residual capacity

Functional residual capacity (FRC) is defined as the volume of air remaining in the lungs at the end of expiration. During MV, increasing PEEP maintains an additional lung volume above FRC, commonly referred to as dynamic FRC (dFRC). This increase in lung volume can enhance oxygenation and improve the benefits of MV; however, excessive PEEP may increase the risk of lung overdistension and overstretching (Sundaresan et al., 2011).

### 4.4 Peak inspiration pressure and peak inspiration volume

Peak Inspiration Pressure (PIP) is defined as the maximum airway pressure reached during the inspiration phase of the ventilation cycle, whereas, Peak Inspiration Volume (PIV) represents the maximum lung volume attained during inspiration. Both PIP and PIV should be measured and monitored continuously during MV (Sun et al., 2022).

### 4.5 Airway resistance

Airway resistance of the respiratory system (R_rs_) describes the opposition to airflow during MV and reflects an important aspect of respiratory system mechanism. Changes in airway resistance can provide valuable insight into respiratory system function and potential airway obstruction during MV (Arunachalam et al., 2020).

### 4.6 Volume control ventilation and pressure control ventilation

MV typically supports patients using either Volume Control Ventilation (VCV) mode or Pressure Control Ventilation (PCV) mode. The VCV keeps the preset volume constant while the pressure can change, but PCV keeps the preset pressure constant as the volume can change according to lung status (Sun et al., 2022).

## 5 SUMMARY OF PREVIOUS STUDIES USING SCLM

This section summarizes prior studies that employed the single compartment lung model (SCLM) to assess and simulate lung mechanics in various clinical contexts. In general, these studies applied the SCLM using data collected from either spontaneously breathing (SB) patients or sedated/non‐SB patients undergoing mechanical ventilation (MV) across different ventilation modes such as VCV and PCV.

Table 1 provides a consolidated overview of 31 studies, outlining each study's focus, key findings, reported advantages, and limitations. The studies vary in terms of objectives, modeling assumptions, and clinical contexts, such as ARDS, COPD, or pediatric applications. To make this work more beneficial, the previous studies were classified according to accuracy metrics as presented in the next section.

**TABLE 1:** Summary of 31 studies applying the Single‐Compartment Lung Model (SCLM) in mechanical ventilation research.

References	Study focus	Key findings	Advantages	Limitations
Chiew et al. (2011)	Dynamic E_drs_ for ARDS at optimal PEEP	Estimated E_drs_ with varying PEEP; linked WOB to elastance	Reduced lung injury through optimized PEEP	Limited to ARDS with non‐SB; Resistance (R) assumed constant, which may affect generalizability to SB or variable‐resistance populations
Kim et al. (2018)	Premature infants	Introduced endotracheal tube (ETT) pressure term into infant MV model	Captures physiological characteristics of preterm lungs	Limited to non‐SB patients; small sample size reduces confidence in broader applicability
Knörzer et al. (2014)	End‐expiratory volume factor α	Optimized E_drs_ using the α factor	Supports ICU decision‐making	Assumes constant resistance; applicability to SB patients and other pathologies not validated
Chiew, Pretty, Shaw, et al. (2015)	E vs. PEEP relation	Explored the relationship between elastance (E), Work of Breathing (WOB), and dFRC during PEEP changes	Balances recruitment and risk of lung injury	SB cases excluded; findings may not generalize to spontaneously breathing populations
Arunachalam et al. (2020)	SB effort modeling	Integrated Gaussian Effort Model (GEM) into SCLM for predicting E and R	Accounts for spontaneous breathing (SB) effort	Limited to VCV mode; results may differ under PCV or mixed modes
Sun et al. (2022)	Virtual model for VCV/PCV with PEEP variation	Validated under PEEP changes; predicted elastance, resistance, PIP, and PIV	Extended findings of (Wong et al., 2022); added new analysis; evaluated prediction metrics	Requires additional validation across diverse MV settings for clinical generalization
Van Drunen, Chiew, Zhao, et al. (2013)	Dynamic elastance map	Visualized E_drs_ variation with PEEP changes	Tailored for ARDS patient assessment	Limited to ARDS patients; Simplified model may not capture variability in other pathologies or SB patients
Morton, Knopp, Docherty, et al. (2018)	Functional capacity (dFRC)	Estimated PIP and dFRC	Supports optimal PEEP selection	Ignores nonlinear flow; limited to non‐SB patients, which may affect extrapolation to SB or different MV modes
Damanhuri et al. (2017)	Negative elastance in SB	Modeled the triggering effect of SB to estimate negative elastance (E)	Enables real‐time detection of spontaneous effort	Requires further validation; limited sample and MV mode, affecting broader generalizability
Chiew, Pretty, Docherty, et al. (2015)	Dynamic elastance (E_drs_) of SB	Decomposed E_drs_ into lung, chest wall, and demand elastance components	Serves as an indicator of ARDS severity	PEEP effect not considered; results may differ under varying PEEP or ventilation modes
Damanhuri et al. (2016)	SB pressure reconstruction	Estimated dynamic respiratory elastance (E_drs_) with M‐wave correction	Accurate estimation of E_drs_	Assumed constant resistance; applicability to non‐SB patients or variable resistance populations is limited
Howe et al. (2018)	Inspiratory from expiratory data	Proposed a method to estimate inspiratory elastance using expiratory phase data	Reuses previously neglected data	Requires real‐time verification; may not generalize to all MV modes or disease conditions
Lerios et al. (2024)	SCLM vs. Nonlinear modeling for COPD	Resistance (R) estimation improved using nonlinear model	Large patient dataset; new validation tools were added	Requires validation for other diseases, COPD‐focused results may not apply to ARDS or COVID‐19 patients
Lerios et al. (2023)	Linear SCLM vs. Nonlinear for COPD (small dataset)	Similar outcomes to (Howe et al., 2018)	Applicable in COPD care	Small sample size (7 subjects); reduces confidence in broader generalization
Hennigs et al. (2023)	COPD with spontaneous breathing (SB)	Lung mechanics were estimated	Simple and solvable approach	Limited to COPD; requires fuzzy logic/tools and further validation to generalize
Merrath et al. (2023)	Simulation of respiratory system mechanics during MV (age, height, and sex considered)	Lung mechanics were modeled	Ability to provide a realistic simulation environment for various patient profiles	Requires validation for additional diseases and SB patients
Avilés‐Rojas & Hurtado (2022)	Finite‐Element Model (FEM) Development	Validated SCLM and FEM to estimate lung elastance (E) and resistance (R)	Simulates VCV/PCV	SB cases not investigated; generalization to spontaneous breathing patients is uncertain
Wong et al. (2022)	Co‐ventilation for two patients	Simulated MV delivery to two patients during a pandemic	Safe simulation for patient pairing	No real‐world data validation; applicability to clinical scenarios limited
Sun et al. (2021)	Virtual model for VCV/PCV considering PEEP variation	Validated under PEEP changes; predicted E, R, PIP, and PIV across PEEP levels	Simulated both VCV and PCV modes	Model remains simplistic; real patient variability not fully captured
Howe et al. (2020)	Expiratory‐based elastance	Compared expiratory elastance with inspiratory elastance	Extracts useful data from expiratory phase	Requires PCV validation; small patient sample reduces reliability
Morton et al. (2020)	Virtual model for PCV	Estimated PIV, E during PCV	Accurate for delivered volume and lung mechanics	Small samples; not validated for SB patients, limiting generalizability
Arunachalam et al. (2020b)	MV virtual protocol (EISEF)	Proposed a five‐stage protocol (EISEF) for MV parameter setting	Structured approach for optimizing MV	Limited to VCV only; applicability to other modes uncertain
Morton, Knopp, Chase, et al. (2018)	Elastance at optimal PEEP	Estimated elastance as a function of volume and pressure at optimal PEEP	High predictive accuracy	Ignores nonlinear flow variations; may affect predictions in complex MV scenarios
Morton, Dickson, Chase, et al. (2018)	Virtual model considering PEEP variability	Predicted PIP, E, and R	Minimizes risk of lung over‐distension	No validation for PCV or SB cases; model simplicity may limit real‐world applicability
Chiew et al. (2018)	Iterative Pressure Reconstruction (IPR) method for SB asynchrony	Introduced MAsyn metric for pressure reconstruction and elastance estimation	Enhances accuracy of lung mechanics modeling in SB	Limited to VCV mode; may not generalize to PCV or mixed‐mode patients
Laufer et al. (2017)	PEEP maneuver study	Estimated elastance before and after PEEP changes	Assists in diagnosing lung status	Requires broader validation across disease conditions; SB and non‐ARDS patients not included
Redmond et al. (2017)	Variable resistance modeling	Modeled resistance (R) as a function of pressure	Captures airway resistance variability	Pressure fluctuations may affect accuracy; limited clinical validation
Szlavecz et al. (2014)	Bedside real‐time tool	Used CURE software o estimate elastance (E)	Clinical utility and training support	Weak performance in SB and PCV modes; results may not generalize
Kannangara et al. (2016)	PCV considering asynchrony magnitude	Applied flow interpolation to estimate E under SB asynchrony	Enhances MV performance for varying SB efforts	Limited to PCV mode; may not generalize to VCV or mixed ventilation
Van Drunen, Chase, Chiew, et al. (2013)	dFRC estimation	Provided a noninvasive alternative to CT/EIT methods for estimating dFRC	Useful ICU tool for monitoring dFRC	Limited to non‐SB patients; validation required for other MV modes
Van Drunen, Chiew, Chase, et al. (2013)	K parameter for expiration	Linked K parameter to lung stiffness for elastance estimation	Tracks lung condition dynamically	Needs validation in ARDS patients with SB; limited generalizability

## 6 ACCURACY METRICS USED IN SCLM ASSESSMENT

Several studies have employed common statistical metrics to assess the accuracy and robustness of SCLMs. This section highlights the key metrics used, without elaborating on basic statistical definitions.

### 6.1 Null hypothesis

This method is often used to assess whether a change in model parameters, for example, PEEP leads to a statistically significant impact on model outputs, for example, respiratory system elastance (E_rs_) (Newbold et al., 2013; Van Drunen, Chiew, Zhao, et al., 2013). If the resulting *p* value is below a defined threshold (commonly 0.05), the null hypothesis is rejected, suggesting a meaningful effect. Table 2 highlights several studies that adopted this approach.

**TABLE 2:** Studies using the single‐compartment lung model (SCLM) and null hypothesis testing as an evaluation metric.

References	Dataset/mode	Study outcome assessment	Metric type	*p* Value
Arunachalam et al. (2020)	8 SB patients (VCV)	SCLM outperformed existing models in spontaneous breathing (SB) cases	Null Hypothesis	<0.005
Van Drunen, Chiew, Zhao, et al. (2013)	Sedated patients (VCV)	E_drs_ varied significantly with PEEP	Null Hypothesis	<0.005
Chiew, Pretty, Docherty, et al. (2015)	22 SB patients (PCV)	NAVA median E_drs_ significantly different from PS E_drs_	Null Hypothesis	<0.05
Lerios et al. (2024)	100 patients (VCV)	Nonlinear model significantly improved resistance (R) estimation in COPD patients	Null Hypothesis	<0.0001
Chiew et al. (2018)	3 SB patients (VCV)	Statistically significant difference between reconstructed and original pressure	Null Hypothesis	<0.05
Laufer et al. (2017)	28 ARDS patients (VCV)	Elastance (E) significantly differed between ARDS and healthy subjects	Null Hypothesis	<0.05
Kannangara et al. (2016)	6 SB patients (PCV)	Reconstruction improved elastance (E) estimation	Null Hypothesis	<0.05

### 6.2 Descriptive metrics

Tools such as the interquartile range (IQR) and median values are also used to assess data variability and distribution, particularly when evaluating model behavior across different ventilator settings.

Tables 2, 3, 4 summarize representative works that utilized these metrics for SCLM assessment (Arunachalam et al., 2020; Chiew, Pretty, Docherty, et al., 2015; Damanhuri et al., 2017; Morton, Knopp, Docherty, et al., 2018).

### 6.3 Absolute percentage error (fitting error)

Frequently referred to as fitting error, this metric quantifies the percentage difference between estimated and true values, thus, smaller errors imply better model accuracy. Other related indicators, such as Mean Absolute Deviation (MAD) and Robust Coefficient of Variation (RCV), have been used less commonly but serve a similar purpose (Damanhuri et al., 2016; Mishra et al., 2012). Table 3 highlights studies that employed fitting error for accuracy assessment.

**TABLE 3:** Studies using the single‐compartment lung model (SCLM) evaluated using fitting error.

References	Dataset/mode	Study outcome assessment	Metric type	Error percent
Chiew et al. (2011)	10 ARDS patients (VCV)	Median E_drs_ error = 0.9%	Fitting error	0.9%
Kim et al. (2018)	10 infants (VCV)	E and R error = 5.7%	Fitting error	5.7%
Arunachalam et al. (2020)	8 SB patients (VCV)	Median error < 10% for airway pressure (P_aw_), elastance (E), and resistance (R)	Fitting error	<10%
Morton, Knopp, Docherty, et al. (2018)	4 ARDS patients (VCV)	PIP prediction error = 1%	Fitting Error	1%
Damanhuri et al. (2016)	5 SB patients (VCV)	RCV variation: 0.04 vs. 0.05	Variation Index	0.04/0.05
Wong et al. (2022)	4 patients (PCV)	Vt prediction error <0.95%	Fitting Error	<0.95%
Morton et al. (2020)	5 patients (PCV)	PIV prediction error = 10.6%	Fitting Error	10.6%
Morton, Knopp, Chase, et al. (2018)	3 ARDS patients (VCV)	PIP prediction error = 10%	Fitting Error	10%
Morton, Knopp, Chase, et al. (2018)	4 sedated patients (VCV)	E and PIP error < 10%	Fitting Error	<10%
Chiew et al. (2018)	3 SB patients (VCV)	Reconstruction error = 3.8%	Fitting Error	3.8%
Kannangara et al. (2016)	6 SB patients (PCV)	Mean Absolute Deviation (MAD) applied for E estimation	MAD	Improved
Van Drunen, Chiew, Chase, et al. (2013)	Sedated patients (VCV)	Fitting error < 10%	Fitting Error	<10%

### 6.4 Coefficient of determination (*R* ^2^)

*R* ^2^ reflects the proportion of variance in observed data explained by the model. Higher *R* ^2^ values suggest better predictive performance. Table 4 presents several works which used the correlation coefficient.

**TABLE 4:** Studies using the single‐compartment lung model (SCLM) evaluated using the coefficient of determination (*R* ^2^) or correlation coefficient (*r*).

References	Dataset/mode	Study outcome assessment	Metric type	*R* ^2^/*r*
Chiew, Pretty, Shaw, et al. (2015)	ARDS patients (VCV)	Elastance (E) vs. Work of Breathing (WOB) *r* = 0.62; E vs. dFRC *r* = −0.62	Pearson r	±0.62
Sun et al. (2022)	36 patients (VCV + PCV)	PIP *R* ^2^ = 0.94; PIV *R* ^2^ = 0.74	*R* ^2^ (Prediction)	0.94 / 0.74
Howe et al. (2018)	8 sedated patients (VCV)	Strong correlation between inspiratory and expiratory elastance (E)	*R* ^2^ (Comparison)	0.94
Sun et al. (2021)	36 patients (VCV + PCV)	Replicated findings as in study (Sundaresan et al., 2011)	*R* ^2^ (Prediction)	0.94/0.74
Howe et al. (2020)	8 patients (VCV)	Comparison of inspiratory vs. expiratory elastance	*R* ^2^ (Comparison)	0.94
Van Drunen, Chase, Chiew, et al. (2013)	10 sedated patients (VCV)	dFRC prediction compared with clinical data	*R* ^2^ (Prediction)	0.415

Table 2 presents studies that used null hypothesis significance testing (*p* values) to support their findings statistically.

Table 3 lists studies that reported fitting error as a quantitative indicator of model accuracy.

Table 4 highlights studies that employed coefficient of determination (*R* ^2^) or correlation coefficients to assess predictive performance.

Table 5 includes studies that used qualitative or descriptive approaches without reporting formal statistical evaluation.

**TABLE 5:** Single‐compartment lung model (SCLM) studies without formal statistical evaluation.

References	Dataset/mode	Study outcome	Remarks
Knörzer et al. (2014)	10 ARDS patients (VCV)	Optimized E_drs_ via α parameter	Visual interpretation
Damanhuri et al. (2017)	5 SB patients (VCV)	Detected negative elastance during spontaneous breathing	Qualitative only
Lerios et al. (2023)	7 patients COPD	Compared SCLM vs. nonlinear models	No formal metric
Hennigs et al. (2023)	SB COPD (PCV)	Modeled pressure‐volume loops and elastance/resistance (E/R) curves	Visual analysis only
Merrath et al. (2023)	SB MV	Vt matched literature values	Initial test
Avilés‐Rojas & Hurtado (2022)	VCV + PCV	Simulated ventilation using finite element lung model	Simulation only
Arunachalam et al. (2020b)	3 patients VCV	Suggested MV protocol	Simulation only
Redmond et al. (2017)	25 mixed patients	Developed pressure‐based variable resistance (R) model	No *p* value or *R* ^2^
Szlavecz et al. (2014)	Non‐SB (VCV)	Used CURE software for bedside lung function tracking	Software evaluation

This structured classification provides a clearer perspective on how the SCLM has been evaluated across the literature and highlights both methodological diversity and recurring limitations.

## 7 RESULTS AND DISCUSSION

In this study, we reviewed and analyzed previous works that applied the SCLM, as summarized in Table 1. The dominant trend observed across the literature is the use of SCLM to estimate lung elastance, a key parameter reflecting lung functionality and patient response to MV. Most studies focused on optimizing MV settings, particularly PEEP and PIP, to improve patient outcomes in both sedated and SB patients. The widespread use of VCV as the mode of MV further reinforced a preference for stable experimental settings. Despite these commonalities, there are notable inconsistencies. For instance, while some studies explored dynamic functional residual capacity (dFRC) and airway resistance, these efforts were rare and not systematically validated. The limited use of expiration data in previous studies reflects a notable gap in current modeling approaches. Expanding on this aspect could lead to a more balanced and accurate representation of respiratory mechanics.

Various metrics were used to assess model accuracy and clinical relevance, including null hypothesis testing (Table 2), fitting error (Table 3), and *R* ^2^‐based analysis (Table 4). These evaluation approaches were explained in Section 4.

However, the choice of evaluation metrics varied significantly among studies and often lacked justification or comparative analysis. This inconsistency hampers the ability to compare findings across studies and represents a significant methodological gap in the literature. Furthermore, some works (as shown in Table 5) did not employ any formal statistical evaluation, further limiting the interpretability and clinical relevance of their findings.

All reviewed studies implemented the SCLM depending on pressure and flow data as model inputs. Nevertheless, Ng et al. (2020) stated that collecting both data types increases system and clinical burden. Therefore, future research could explore simplified implementations using a single input (e.g., pressure or flow) to reduce complexity and broaden clinical applicability. This review highlights several key insights from studies employing the SCLM framework. While the estimation of lung elastance remains the primary and clinically valuable focus, other parameters such as airway resistance and dFRC have not been sufficiently explored, despite their importance in conditions like ARDS and COPD. Additionally, there is a notable lack of standardization in the evaluation metrics applied across studies, with limited justification provided for metric selection, which hinders meaningful comparison and synthesis of findings.

The absence of clear data collection protocols, particularly regarding patient status (sedated versus spontaneously breathing) and ventilation mode (VCV vs. PCV) limits the generalizability of reported findings. This lack of methodological transparency remains a recurring issue across prior studies. In addition, the expiration phase, which could offer valuable insights and simplify model inputs, remains an underutilized component in SCLM‐based modeling. Addressing these methodological gaps and inconsistencies in future research through comparative metric studies, simplified input modeling, and disease‐specific validation will improve the reliability and clinical utility of SCLM‐based approaches in mechanical ventilation.

Moreover, the COVID‐19 pandemic underscored the urgent need for simple and real‐time respiratory models such as the SCLM to support clinical decision‐making in critically ill patients.

In COVID‐19‐associated ARDS, continuous monitoring of lung compliance and elastance became essential for optimizing ventilator settings and minimizing the risk of ventilator‐induced lung injury (VILI). Despite this clear clinical relevance, few studies have directly applied the SCLM framework to COVID‐19 patient data. This represents a notable gap in the literature and highlights an important opportunity for future research to validate and adapt SCLM‐based models for pandemic and emergency healthcare settings.

## 8 CONCLUSION

Lung modeling is used to mimic lung behavior to solve clinical problems. It also can be done to study lung behavior during MV, which is important to treat serious lung diseases such as COVID‐19, etc. This paper provided an overview of various studies that aimed to develop SCLM in clinical practices for describing lung functionality and accordingly guide clinicians to improve MV therapy. The review showed that most studies focused on estimating lung elastance, while other important parameters like airway resistance (R_rs_) and dFRC were less explored. It also identified variations in methodologies and evaluation tools used in different studies, which make comparison difficult. Additionally, the use of expiration‐phase data was limited, even though it could help make the model simpler and more accurate. Furthermore, this review acknowledges the potential of incorporating fractal theory to account for the multiscale structural complexity of lung anatomy. Integrating such complexity‐based simulations is essential for achieving patient‐specific care in both restrictive and obstructive physiologies. This review highlights the need for clearer standards and more practical models that can be used easily in real clinical situations, especially during emergencies like COVID‐19. These findings can guide future works to make SCLM‐based models more useful for improving patient care during MV.

## AUTHOR CONTRIBUTIONS

**Husam Y. Al‐Hetari:** Conceptualization; methodology; software. **Mahmoud A. Al‐Rumaima:** Investigation; validation. **Hamdi H. Ghazi:** Formal analysis; visualization. **Noman Q. Al‐Naggar:** Project administration; validation. **E. A. Ali:** Formal analysis; investigation; visualization. **Abdu Alameri:** Data curation; supervision.

## FUNDING INFORMATION

This research received no external funding.

## CONFLICT OF INTEREST STATEMENT

The authors declare that they have no known competing financial interests or personal relationships that could have appeared to influence the work reported in this paper.

## DISCLOSURE

Artificial intelligence tools were used solely to assist in rephrasing some sentences in this manuscript. The authors take full responsibility for the content.

## ETHICS STATEMENT

Not applicable.
